# Correction: “Distinguishing Phylogenetic Level-2 Networks with Quartets and Inter-Taxon Quartet Distances”

**DOI:** 10.1007/s11538-025-01564-5

**Published:** 2026-01-14

**Authors:** Niels Holtgrefe, Elizabeth S. Allman, Hector Baños, Leo van Iersel, Vincent Moulton, John A. Rhodes, Kristina Wicke

**Affiliations:** 1 Delft Institute of Applied Mathematics, Delft University of Technology, Delft, The Netherlands; 2 Department of Mathematics and Statistics, University of Alaska Fairbanks, Fairbanks, AK USA; 3 Department of Mathematics, California State University San Bernardino, San Bernardino, CA USA; 4 School of Computing Sciences, University of East Anglia, Norwich, UK; 5 Department of Mathematical Sciences, New Jersey Institute of Technology, Newark, NJ USA

Correction to: Bulletin of Mathematical Biology (2025) 87:168 10.1007/s11538-025-01549-4

The authors regret that Fig. 12b was inadvertently omitted from the published version of this article due to a production error. This correction does not affect the results, analysis, or conclusions presented in the original publication.

**Fig. 12:**
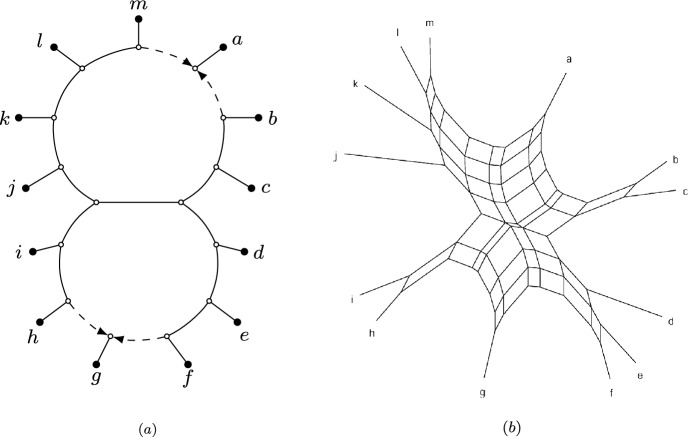
Left: An outer-labeled planar, galled, level-2, semi-directed bloblet network N on leaf set X = {a,…, m}. Right: The splits graph of the pairwise NANUQ distances $d_N$of the network N, obtained with Neighbor- Net (Bryant and Moulton 2004)

The complete version of Fig. 12b is provided here.

The original article has been corrected.

